# Association between hospital acquired disability and post-discharge mortality in patients after living donor liver transplantation

**DOI:** 10.1186/s12893-022-01896-2

**Published:** 2022-12-29

**Authors:** Masatoshi Hanada, Masaaki Hidaka, Akihiko Soyama, Takayuki Tanaka, Takanobu Hara, Hajime Matsushima, Masafumi Haraguchi, Mineaki Kitamura, Motohiro Sekino, Masato Oikawa, Hiroki Nagura, Rina Takeuchi, Shuntaro Sato, Hideaki Takahata, Susumu Eguchi, Ryo Kozu

**Affiliations:** 1 Cardiorespiratory Division, Department of Rehabilitation Medicine, Nagasaki University Hospital, 1-7-1 Sakamoto, Nagasaki, 852-8501 Japan; 2 Department of Cardiopulmonary Rehabilitation Science, Nagasaki University Graduate School of Biomedical Sciences, Nagasaki, Japan; 3 Department of Surgery, Nagasaki University Graduate School of Biomedical Sciences, Nagasaki, Japan; 4 Department of Gastroenterology and Hepatology, Nagasaki University Graduate School of Biomedical Sciences, Nagasaki, Japan; 5 Department of Nephrology, Nagasaki University Graduate School of Biomedical Sciences, Nagasaki, Japan; 6 Division of Intensive Care, Nagasaki University Hospital, Nagasaki, Japan; 7 Clinical Research Center, Nagasaki University Hospital, Nagasaki, Japan

**Keywords:** Activities of daily living, Early mobilization, Living donor liver transplantation, Mortality

## Abstract

**Background:**

Hospital-acquired disability (HAD) in patients who undergo living donor liver transplantation (LDLT) is expected to worsen physical functions due to inactivity during hospitalization. The aim of this study was to explore whether a decline in activities of daily living from hospital admission to discharge is associated with prognosis in LDLT patients, who once discharged from a hospital.

**Methods:**

We retrospectively examined the relationship between HAD and prognosis in 135 patients who underwent LDLT from June 2008 to June 2018, and discharged from hospital once. HAD was defined as a decline of over 5 points in the Barthel Index as an activity of daily living assessment. Additionally, LDLT patients were classified into four groups: low or high skeletal muscle index (SMI) and HAD or non-HAD. Univariate and multivariate Cox proportional hazard models were used to evaluate the association between HAD and survival.

**Results:**

HAD was identified in 47 LDLT patients (34.8%). The HAD group had a significantly higher all-cause mortality than the non-HAD group (log-rank: p < 0.001), and in the HAD/low SMI group, all-cause mortality was highest between the groups (log-rank: p < 0.001). In multivariable analysis, HAD was an independent risk factor for all-cause mortality (hazard ratio [HR]: 16.54; P < 0.001) and HAD/low SMI group (HR: 16.82; P = 0.002)**.**

**Conclusion:**

HAD was identified as an independent risk factor for all-cause mortality suggesting that it could be a key component in determining prognosis after LDLT. Future larger-scale studies are needed to consider the overall new strategy of perioperative rehabilitation, including enhancement of preoperative physiotherapy programs to improve physical function.

**Supplementary Information:**

The online version contains supplementary material available at 10.1186/s12893-022-01896-2.

## Introduction

Since the first successful liver transplantation performed by Starzl et al. in 1967 [1], in patients with end-stage liver disease, liver transplantation is a life-saving last treatment measure and proven intervention [2, 3]. One and five years survival rates of the patients following liver transplantation were 85%–86% and 68%–74%, respectively [4].

In Japan, only a few liver donors were found while accessing deceased donors. Therefore, in order to facilitate organ donation, the only option left is to choose living donor liver transplantation (LDLT) [5]. In the registry data by the Japanese Liver Transplantation Society, the number of LDLT performed was approximately 400 cases per year [6].

Patients with chronic cirrhosis often develop sarcopenia as loss of skeletal muscle mass and strength due to protein-energy malnutrition and decreased physical activity [7, 8]. These problems have led to decreased quality of life and increased mortality rate, infection, and postoperative complications in patients with cirrhosis [9]. In addition, the disease progression due to the long waiting period, and inactive patients awaiting liver transplantation further increases the risk of sarcopenia progression [10]. Tandon et al.[11] showed that approximately 40% of patients awaiting liver transplantation have sarcopenia. Therefore, it is important to prevent sarcopenia to potentially improve the overall treatment of patients undergoing LDLT [12]. However, previous studies have reported that sarcopenia is not related to prognosis of LDLT [13, 14]. Therefore, the association between sarcopenia and prognosis in LDLT patients is still controversial and far from being elucidated.

Recently, hospital-acquired disability (HAD), a decrease in physical function associated with hospitalization, has been regarded as a major problem [15]. Cheville et al. [16] showed the staggering magnitude of hospitalization-associated disablement which has been recognized for over a decade. Approximately 30% of hospitalized older adults develop a new disability until discharge, which increases the risk for readmission, institutionalization, and mortality [17]. Generally, in-patients are treated with the acute medical and surgery issues priority, due to which these patients decline loss of skeletal muscle function and, activities of daily living (ADL). At the time of discharge these patients often develop a major new disability that was not present before the onset of acute illness [18] Almost all preoperative LDLT patients have expected comorbid HAD, because of their severe condition.

Physiotherapy plays a major role in perioperative management of LDLT and is the most important prevention strategy for HAD. Peri-operative physiotherapy, including early mobilization in LDLT patients, has been considered essential for the prevention of various complications during the postoperative period [19]. However, mobilization after surgery can be often limited because recovery in several LDLT patients is dependent on the liver graft recovery [20]. Although LDLT patients easily develop HAD, the short-and long-term influences of HAD in these patients remain unknown.

There is a potential to develop new prevention strategies for HAD in LDLT patients which can reduce the long-term effects of physical dysfunction and health care costs. We hypothesized that HAD would deteriorate prognosis compared to a control group in LDLT patients. Therefore, the present study aimed to investigate the prevalence of HAD and the association of prognosis after hospitalization in patients undergoing LDLT.

## Materials and methods

### Study design and study population

This was a single-center, retrospective cohort study conducted at Nagasaki University Hospital that enrolled patients who underwent LDLT. This study was approved by the Human Ethics Review Committee of Nagasaki University Hospital (Approval number: 20012022), and was carried out according to the Declaration of Helsinki. Informed consent was obtained in the form of an opt-out on the website of Nagasaki University Hospital.

Patients who underwent LDLT were investigated between June 2008 and June 2018. They were screened for eligibility criteria which included patients ≥ 20 years of age, undergoing planned surgery for LDLT, and discharged at home, or transferred to another hospital for rehabilitation after LDLT. Patients who had comorbid conditions that could affect exercise performance (e.g., musculoskeletal or neurological impairment), died after LDLT without any discharge, and underwent re-transplantation were excluded.

## Measurements

### Definition of hospital-acquired disability (HAD)

Activities of daily living (ADL) were evaluated using the Barthel Index [21]. The scale evaluates 10 fundamental daily activities (feeding, bathing, grooming, dressing, bowels, bladder, toilet use, transfer, mobility, and climbing stairs). Each of the 10 activities was classified as unable, mild (5 points), moderate (10 points), and independent (15 points). The total score was used for the analysis. In accordance with previous studies, the mean change in Barthel Index score was a decrease of 4.8–5 points from the time of pre-hospitalization to hospital discharge [22, 23]. Therefore, according to the previous study, HAD was defined as decrease of at least 5-points on the Barthel Index [23]. We compared between HAD and non-HAD groups.

### The geriatric nutritional risk index (GNRI)

Preoperative nutritional status using GNRI has been associated with the postoperative course in abdominal surgery [24]. The GNRI can be calculated as follows: [1.489 × albumin (g/dL)] + [41.7 × (weight/ideal weight)], where the formula for the ideal body weight is: [height (m^2^) × 22 (body mass index: BMI)] [25]. The GNRI has four grades of nutrition-related risk and is classified as high risk (< 82), moderate risk (82 to < 92), low risk (92–98), and no risk (> 98). In accordance with previous studies, we defined low or severe nutrition-related risk (GNRI < 92) or no nutrition-related risk (GNRI ≥ 92) [26, 27].

### Skeletal muscle mass index (SMI)

Abdominal computed tomography (CT) was performed within 1 month before the operation. The sum of cross-sectional areas of the L3 skeletal muscles was calculated using the psoas muscle, lumbar muscle, erector spinae, transversus abdominis muscle, internal and external oblique muscles, and rectus abdominis to assess preoperative abdominal sarcopenia [28]. The L3 level of the skeletal muscle area was defined semi-automatically using SYNAPSE VINCENT™ software (Fujifilm Medical Co., Ltd., Tokyo, Japan), and the muscle area was quantified based on the CT Hounsfield unit (HU) range which was − 29 to + 150 HU. To normalize muscle area at the L3 level, the skeletal muscle index (SMI) was calculated by dividing the total muscle cross-sectional area (cm^2^) by the square of the patient's height (m^2^). According to a previous study, sarcopenia was defined as an L3 muscle index of < 42 cm^2^/m^2^ for men and < 38 cm^2^/m^2^ for women [29].

In the sub-analysis, we considered four subgroups in LDLT patients [low or high skeletal muscle index [SMI] and HAD or non-HAD). HAD/non-HAD and low/high SMI were defined as cut-off points (HAD, BI < 5; non-HAD, BI ≥ 5; and male, low; SMI < 42, high; SMI ≥ 42, female, low; SMI < 38, high; SMI ≥ 38].

### Peri-operative physical therapy program

All patients recruited in the study received routine pre- and postoperative physical therapy. Physical therapy was initiated approximately 7 days preoperatively, and was mainly focused on breathing exercises to prevent postoperative pulmonary complications, and educating about the importance of early mobilization during the postoperative period. Based on a previous study [30], the safety inception criteria for early mobilization were defined as follows: 40 bpm < heart rate < 130 bpm; 90 mmHg < systolic arterial pressure < 200 mmHg; 60 mmHg < mean arterial pressure < 110 mmHg; 5 breaths per minute < respiratory rate < 40breaths per minute, percutaneous oxygen saturation ≥ 90%, and awareness at a sufficient level of consciousness that allowed correct understanding. Postoperatively, physical therapy consisted of early mobilization after surgery, resistance training, and aerobic exercise such as walking or cycling at the gym from postoperative day 1 until discharge. All patients received standard perioperative medical and nursing care.

### Clinical data

Previous medical condition data were collected from the patients’ medical charts. The clinical data including the donor age, recipient age, the Model for End-stage Liver Disease (MELD) score [31], Chronic kidney disease (CKD), estimated glomerular filtration rate (eGFR), type of disease, operative time, the rate of ABO-incompatible recipients, the graft volume/standard liver volume ratio (GW/SLV), and quantity of blood loss during surgery. The CKD was defined as more than 3 months of continuous deterioration of renal function with an eGFR < 60 mL/min/1.73m^2^ [32]. According to the Japanese Society of Nephrology, the eGFR was calculated as 194 × Cr − 1.094 × age − 0.287 for male and the same value × 0.739 for female [33].

### Outcomes and follow-up

The primary outcome was to determine the impact of HAD on overall survival after the discharge of LDLT patients. The secondary outcome was to identify independent factors, such as preoperative muscle mass and HAD, which are associated with overall survival. In addition, a similar consideration was made by dividing the four groups into associations of HAD and SMI.

### Statistical analysis

The Shapiro–Wilk test was used to analyze the normality of the data. Normally distributed data were analyzed using Student’s t-test, non-normally distributed data were analyzed using the Mann–Whitney U test, and categorical data were analyzed using the chi-square test for between-group comparisons. Data are expressed as median (interquartile range [IQR]) or number and percentage of patients. The change in ADL score from pre-hospitalization to hospital discharge was compared using the Wilcoxon signed-rank test. Overall survival was calculated using the Kaplan–Meier method and compared using the log-rank test. Overall survival was censored from the time of operation to death or the last follow-up. We analyzed using similar comparisons in four subgroups (low or high SMI and/or HAD or non-HAD) as subgroup analysis. Univariate and multivariate Cox proportional hazard models were used to identify the prognostic factors of overall survival. Variables with p-values of < 0.20 by the univariable test, were included in the multivariable analysis [34]. Statistical analyses were carried out using JMP software (version 15.0; SAS Institute Japan, Tokyo, Japan).

## Results

### The baseline characteristics and the differences between with or without HAD

**Fig. 1:**
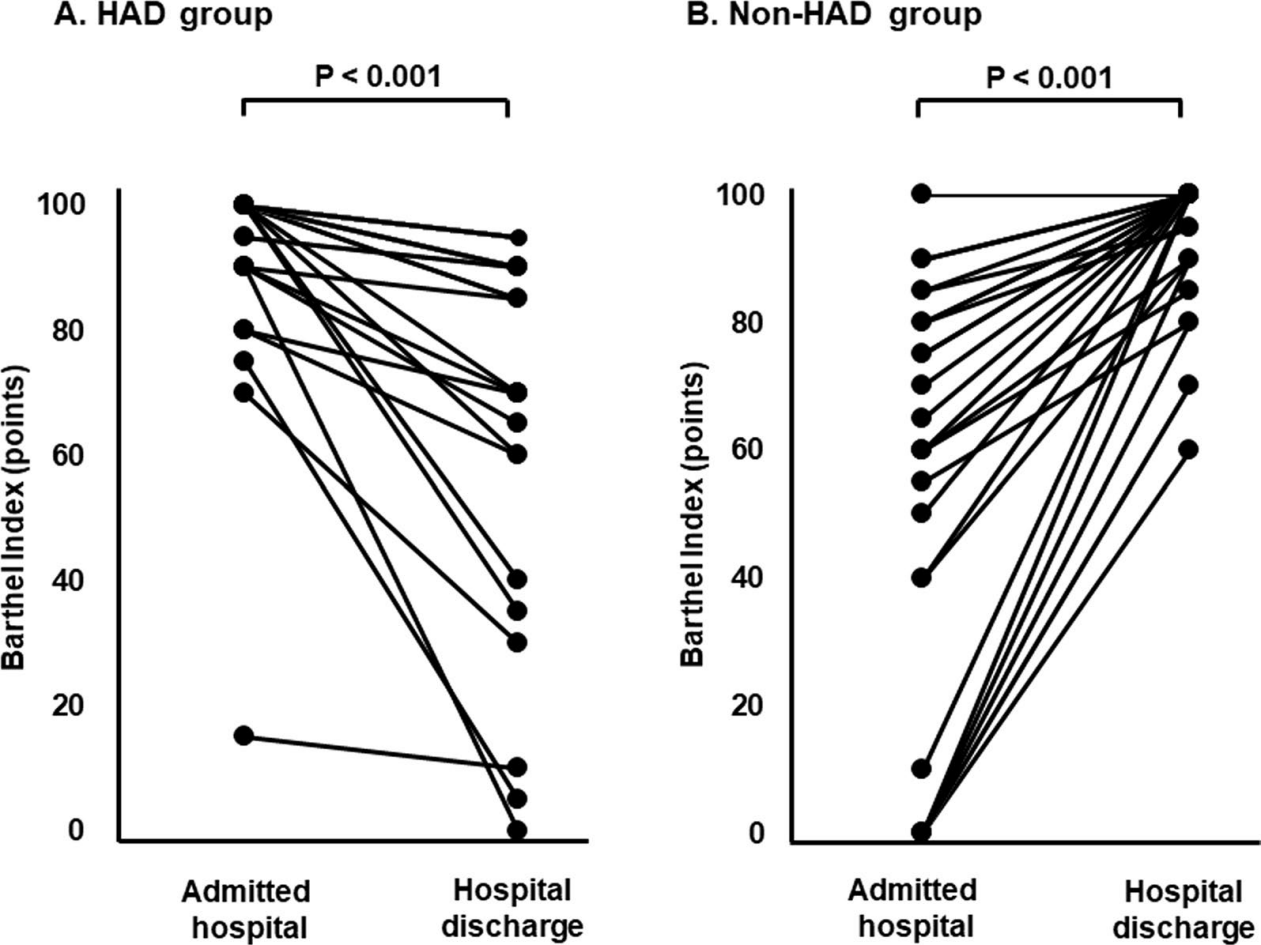
Changes in the Barthel Index from admission to discharge in LDLT patients with HAD group and non-HAD group. *AD* hospital acquired disability; *LDLT* living donor liver transplantation

The eligibility of 191 patients who underwent LDLT was examined in this study. Of these, 56 were excluded (dead during hospitalization, n = 37; re-transplantation, n = 2; pediatric LDLT, n = 4; evaluation refusal, n = 3; and missing data, n = 10). The demographic data, clinical characteristics, and comparisons with or without HAD groups are presented in Table 1. A total of 135 LDLT patients were compared to the HAD group (n = 47, median [IQR]: 57.0 [51.0–65.0] years) or control group (n = 88, median [IQR]: 57.0 [52.0–62.0] years). Although there were no significant differences in the MELD score as the severity of liver disease, it tended to be high in the HAD group. In the HAD group, the operative time was significantly longer than in the control group. CKD as comorbidity, preoperative and postoperative eGFR, operative blood loss, donor age, left lobe graft, GW/SLV, and ABO-incompatibility were not significantly different between the groups. In the non-HAD group, the initial walking day was significantly earlier than in the HAD group (p = 0.013). Hospital length of stay of HAD group was significantly longer (p < 0.001), rate of transfer to hospital was significantly higher than non-HAD group (p = 0.001). Changes in the Barthel Index from admission to discharge in LDLT patients were significantly different in the (A) HAD group and (B) non-HAD group, respectively (p < 0.001) (Fig. 1). In the proportion of dependent ADL score, climbing stairs and bathing activities were higher than other activities.

**Table 1:** Comparison of LDLT patients and surgery characteristics according to allocation group

	Overall (n = 135)	HAD group (n = 47)	Non-HAD group (n = 88)	p-value
Gender, male, n (%)	73 (54.1)	25 (53.2)	48 (54.6)	1.000
Age, year	57.0 (52.0–62.0)	57.0 (51.0–65.0)	57.0 (52.0–62.0)	0.701
BMI, kg/m^2^	19.6 (17.0–22.3)	20.0 (17.0–22.1)	19.5 (16.9–22.5)	0.908
GNRI, points	81.6 (73.7–90.9)	81.6 (73.7–90.3)	81.8 (72.8–91.1)	0.702
< 92	105 (77.8)	38 (80.9)	67 (76.1)	0.665
≥ 92	30 (22.2)	9 (19.2)	21 (23.9)	
Type of disease, n (%)				
Primary biliary cirrhosis	11 (8.1)	6 (12.8)	5 (5.7)	0.557
Primary sclerosing cholangitis	7 (5.2)	3 (6.4)	4 (4.6)	
Hepatitis B virus	15 (11.1)	4 (8.5)	11 (12.5)	
Hepatitis C virus	51 (37.8)	19 (40.4)	32 (36.4)	
Non-B, non-C liver cirrhosis	13 (9.6)	5 (10.6)	8 (9.1)	
Other	38 (28.1)	10 (21.3)	28 (31.8)	
Comorbidity, n (%)				
Hepatocellular carcinoma	54 (40.0)	20 (42.6)	34 (38.6)	0.714
Chronic kidney disease	59 (43.7)	23 (48.9)	36 (40.9)	0.467
Pre-operative eGFR, mL/min/1.73m^2^	66.9 (46.4–86.4)	62.0 (43.3–87.7)	69.8 (49.1–85.6)	0.722
Post-operative eGFR, mL/min/1.73m^2^	62.8 (46.1–74.4)	61.5 (46.2–74.1)	63.5 (45.9–74.4)	0.541
Δ eGFR (admission to discharge), mL/min/1.73m^2^	9.4 (− 10.3 to 20.8)	9.8 (− 10.3 to 30.3)	8.8 (− 10.7 to 18.0)	0.567
MELD score	16.0 (12.0–22.0)	15.0 (12.0–20.0)	17.0 (12.0–22.0)	0.393
Operation time, min	761.0 (701.0–849.0)	798.0 (710.0–925.0)	750.0 (695.0–820.3)	0.036
Operative blood loss, g	5550 (3550–9500)	7000 (3140–12,670)	4935 (3663–8735)	0.223
Donor age, year	33 (27–43)	33 (26.0–42.0)	33 (27.0–45.3)	0.914
Left lobe graft, n (%)	87 (64.4)	31 (66.0)	56 (63.6)	0.470
GW/SLV, %	40.2 (33.7–49.3)	39.7 (33.7–44.5)	41.4 (33.3–51.8)	0.170
ABO-incompatible, n (%)	30 (22.2)	8 (17.0)	22 (25.0)	0.076
SMI, cm^2^/m^2^				
Male, n (%)	44.5 (39.0–48.8)	44.5 (40.8–48.6)	44.4 (37.9–49.1)	0.803
Female, n (%)	41.0 (36.1–47.9)	41. (37.9–49.2)	41.1 (34.9–47.7)	0.354
Initial walking, day	9 (6–15)	13.0 (7.0–19.0)	8.0 (6.0–12.8)	0.013
ICU length of stay, days	5 (4–8)	5.0 (4.0–9.0)	5.0 (4.0–7.8)	0.218
BI at admission, points	100 (90–100)	100 (100–100)	100 (80–100)	0.032
BI at hospital discharge, points	100 (90–100)	90 (70–95)	100 (100–100)	< 0.001
Hospital length of stay, days	48.0 (37.0–67.0)	58.0 (46.0–76.0)	45.0 (36.0–58.8)	< 0.001
Transfer to hospital, n (%)	29 (21.6)	18 (39.1)	11 (12.5)	0.001
Period after discharged from a hospital, months	28.1 (27.6–28.4)	27.8 (27.3–28.1)	28.2 (27.8–28.4)	< 0.001
Cause of death, n (%)	13 (9.6)	10 (21.3)	3 (3.4)	0.189
Factors of graft side	4 (3.0)	4 (8.5)	0	
Factors of recipient side	3 (2.2)	2 (4.3)	1 (1.1)	
Hepatocellular carcinoma recurrence	1 (0.7)	0	1 (1.1)	
Other carcinoma	3 (2.2)	3 (6.4)	0	
Other	2 (1.5)	1 (2.1)	1 (1.1)	

Values were reported as the median and Interquartile range (IQR) or number of subjects and percentage

*BI* Barthel index; *BMI* body mass index; *eGFR* estimated glomerular filtration rate; *GNRI* Geriatric Nutritional Risk Index; *GW*/*SLV*: graft weight/standard liver volume; *HAD* hospital acquired disability; *ICU* intensive care unit; *IQR* interquartile range; *LDLT* living donor liver transplantation; *MELD* score model for end-stage liver disease score; *SMI* skeletal muscle index

The demographic data, clinical characteristics and a comparison of the four subgroups (HAD/non-HAD and/or low/high SMI) are presented in Table 2. Similar results were obtained in the analyses of the baseline characteristic data in the four subgroups. The SMI of the non-HAD/low SMI group was significantly lower than that of the other groups (p < 0.001). Hospital length of stay was significantly longer in the HAD/low SMI group than in the other groups (p < 0.001). In the HAD/high SMI and HAD/low SMI groups, the rate of transfer to hospital was significantly higher than that in the non-HAD/high SMI and non-HAD/low SMI groups (p < 0.01).

**Table 2:** Comparison of LDLT patients and surgery characteristics according to four group

	Non-HAD and high SMI group (n = 58)	Non-HAD and Low SMI group (n = 31)	HAD and high SMI group (n = 34)	HAD and low SMI group (n = 12)	p-value
Gender, male, n (%)	33 (57.0)	15 (48.4)	18 (52.9)	7 (58.3)	0.874
Age, year	57.0 (52.0–63.0)	57.0 (50.0–61.0)	57.0 (52.0–65.0)	56.0 (46.3–63.3)	0.695
BMI, kg/m^2^	20.4 (17.7–23.4)	18.9 (15.8–21.0)	19.9 (17.7–22.1)	19.6 (16.2–22.0)	0.087
GNRI, points	83.8 (75.5–92.2)	78.7 (69.7–85.8)	84.3 (73.6–91.5)	80.7 (75.2–89.1)	0.392
< 92	43 (74.1)	25 (80.7)	27 (79.4)	10 (83.3)	0.835
≥ 92	15 (25.9)	6 (19.4)	7 (20.6)	2 (16.7)	
Type of disease, n (%)					
Primary biliary cirrhosis	4 (6.9)	1 (3.2)	6 (17.7)	0	0.074
Primary sclerosing cholangitis	2 (3.5)	2 (6.5)	1 (2.9)	2 (16.7)	
Hepatitis B Virus	8 (13.8)	3 (9.7)	4 (11.8)	0	
Hepatitis C virus	21 (36.2)	12 (38.7)	12 (35.3)	6 (50.0)	
Non-B, non-C liver cirrhosis	6 (10.3)	2 (6.5)	4 (11.8)	1 (8.3)	
Other	17 (29.3)	11 (35.5)	7 (21.0)	3 (25.0)	
Comorbidity, n (%)					
Hepatocellular carcinoma	25 (43.1)	10 (32.3)	14 (41.8)	5 (41.7)	0.792
MELD score	17.0 (11.0–21.3)	17.0 (12.0–18.0)	15.0 (12.0–18.3)	17.0 (13.0–23.0)	0.796
Operation time, min	752.0 (702.5–829.3)	739.0 (686.0–803.0)	780.5 (706.0–904.0)	849.0 (751.3–962.0)	0.064
Operative blood loss, g	5225 (3463–8627)	4900 (3700–8900)	6300 (2899–12,337)	7848 (4900–13,292.5)	0.248
Donor age, year	31.5 (26.8–40.3)	36.0 (30.0–53.0)	33.0 (25.5–40.3)	34.0 (29.0–45.8)	0.504
Left lobe graft, n (%)	36 (62.1)	21 (67.7)	22 (64.7)	8 (66.7)	0.642
GW/SLV, %	39.7 (33.7–51.6)	42.5 (33.0–52.9)	40.2 (33.5–44.4)	37.8 (33.7–45.3)	0.626
ABO-incompatible, n (%)	11 (19.0)	11 (35.5)	7 (20.1)	1 (8.3)	0.029
SMI, cm^2^/m^2^					
Male, n (%)	47.8 (44.1–55.4)	35.5 (30.7–39.3)	46.6 (43.8–49.6)	37.6 (35.6–41.2)	< 0.001
Female, n (%)	46.7 (43.2–48.9)	33.4 (28.9–36.1)	43.0 (39.9–49.5)	34.5 (32.4–37.1)	< 0.001
Initial walking, day	8.0 (5.0–11.3)	11.0 (7.0–15.0)	13.0 (6.8–19.3)	11.5 (6.3–41.3)	0.052
ICU length of stay, days	5.0 (3.8–7.3)	5.0 (4.0–8.0)	5.5 (4.0–9.0)	5.5 (5.0–15.8)	0.237
BI at admission, points	100 (80–100)	100 (65–100)	100 (95–100)	100 (100–100)	0.119
BI at hospital discharge, points	100 (100–100)	100 (100–100)	90 (70–95)	90 (85–95)	< 0.001
Hospital length of stay, days	42.0 (33.0–52.3)	52.0 (37.0–76.0)	58.0 (46.8–74.5)	58.0 (39.3–99.5)	< 0.001
Transfer to hospital, n (%)	5 (8.6)	6 (19.4)	14 (42.4)	4 (33.3)	< 0.01

Values were reported as the median and Interquartile range (IQR) or number of subjects and percentage

*BI* Barthel index; *BMI* body mass index; *GNRI* Geriatric Nutritional Risk Index; *GW*/*SLV* graft weight/standard liver volume; *HAD* hospital acquired disability; *ICU* intensive care unit; *IQR* interquartile range; *LDLT* living donor liver transplantation; *MELD score* model for end-stage liver disease score; *SMI* skeletal muscle index

### Overall survival analyses according to HAD in patients with LDLT

**Fig. 2:**
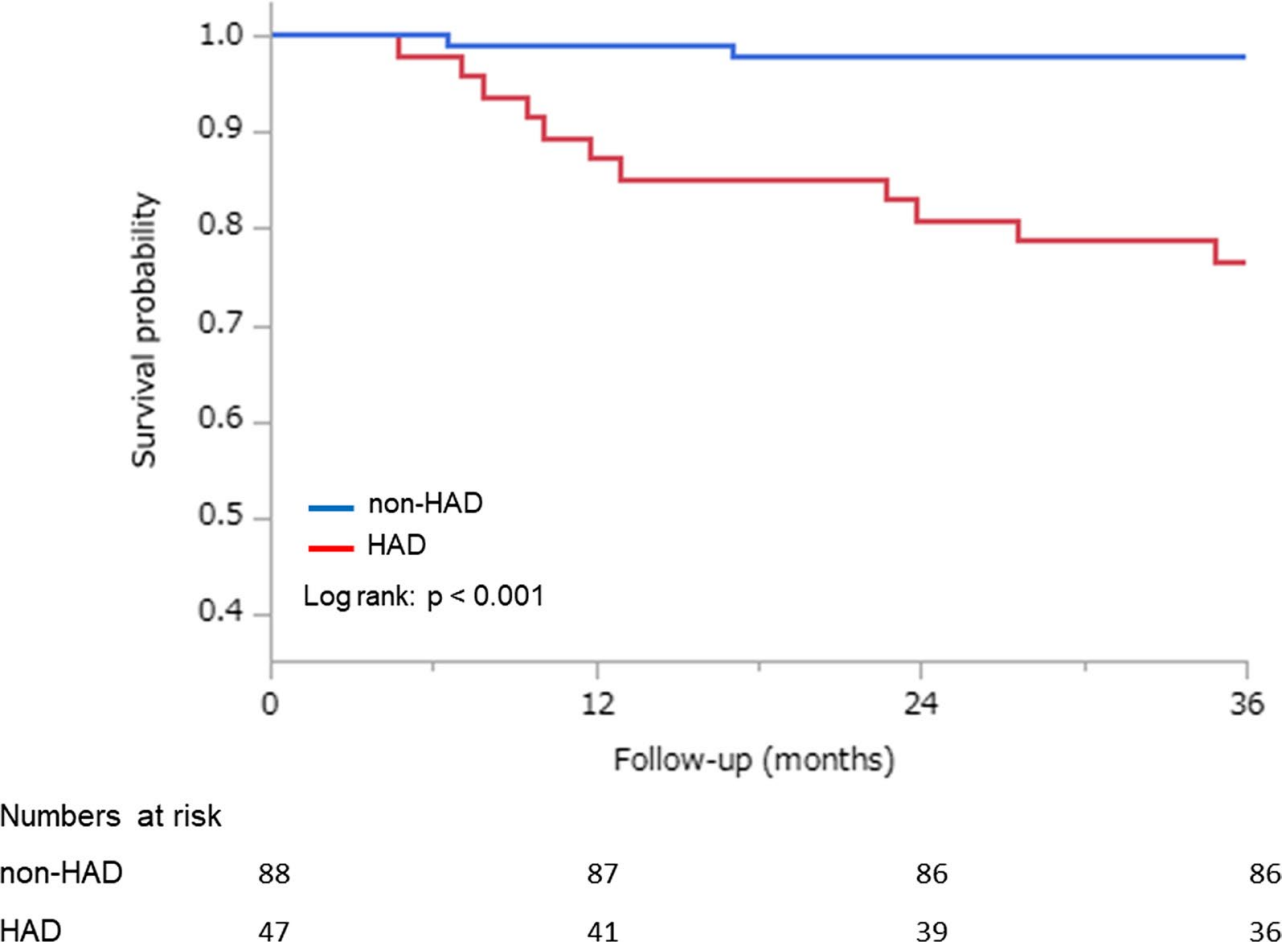
Kaplan–Meier curves of overall survival for HAD and non-HAD in LDLT patients. *HAD* hospital acquired disability; *LDLT* living donor liver transplantation

**Fig. 3:**
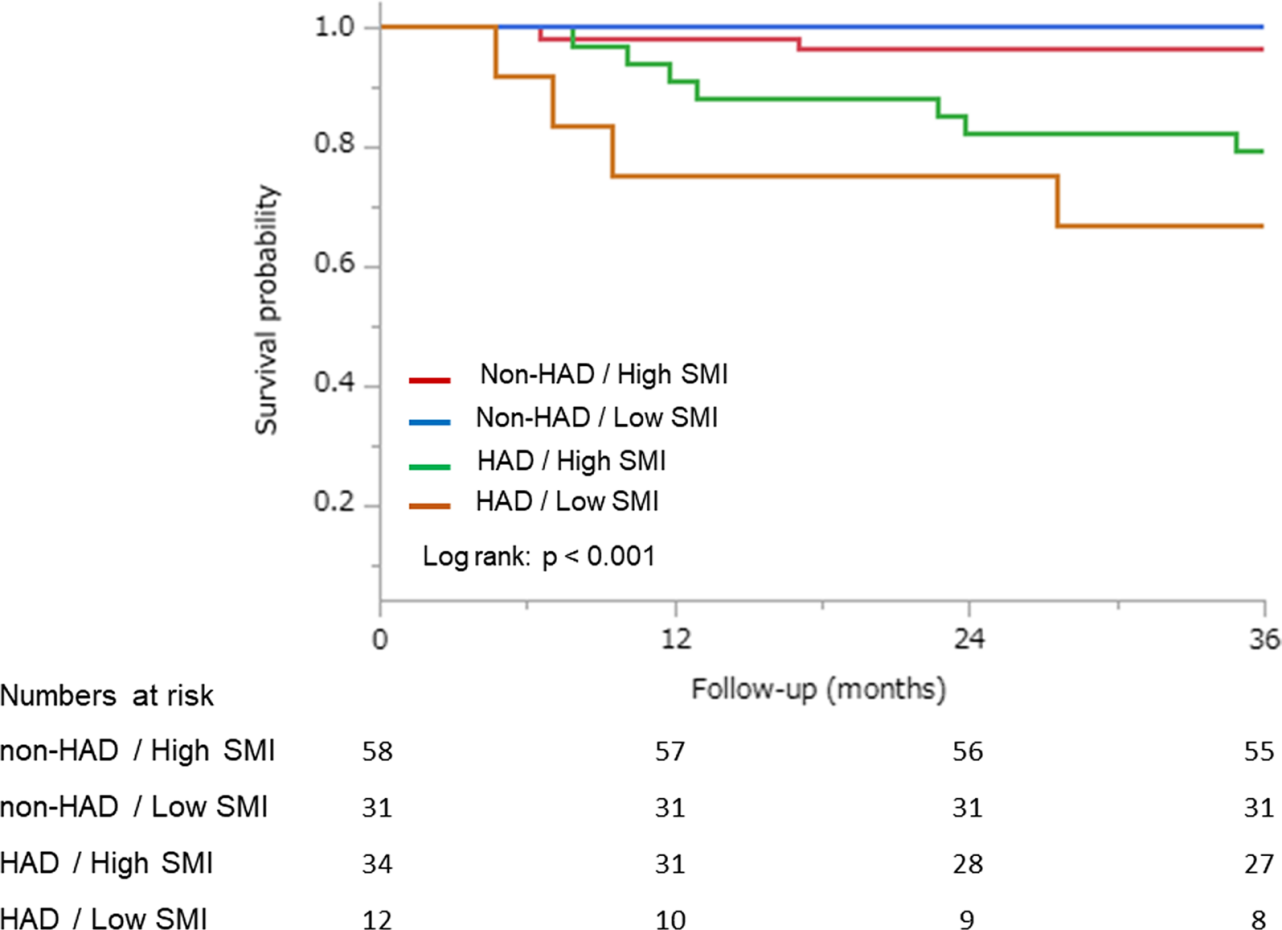
Kaplan–Meier curves of overall survival according to HAD and SMI for four groups in LDLT patients. *HAD* hospital-acquired disability; *LDLT* living donor liver transplantation; *SMI* skeletal muscle index

The median follow-up overall survival of all patients was 6.8 year (IQR, 4.3–9.6 year). A total of 13 (9.6%) deaths occurred during the investigative period. The causes of death in the recipients after LDLT were graft failure (n = 3), infection (n = 2), hepatocellular carcinoma recurrence (n = 1), sepsis (n = 1), other carcinomas (n = 3), post-transplant lymphoproliferative disorders (n = 1), chronic rejection (n = 1), and cerebral hemorrhage (n = 1). Although the cause of death was not significantly different between the groups, factors of graft and recipient side in the HAD group were higher than those in the non-HAD group (Table 1). In the 3-year overall survival, the HAD group had a significantly higher all-cause mortality than the non-HAD group in the Kaplan–Meier survival curves (log-rank: p < 0.001; Fig. 2), and in the HAD/low SMI group, all-cause mortality was highest between the groups (log-rank: p < 0.001; Fig. 3). In addition, we considered a sub-analysis that comparison between low and high SMI groups. As a result, the patients with HAD were no significant differences between low SMI group and high SMI group. LDLT patients with HAD were 20–30% dead in both groups (Additional file 1: Table S1). The cause of death in low SMI group were graft failure, infection, other carcinomas, cerebral hemorrhage, and in high SMI groups, graft failure and other carcinomas had most common (Additional file 2: Table S2).

### The results of the univariable and multivariable analyses for survival and HAD

Univariate analysis identified seven significant prognostic factors for survival: the presence of HAD, MELD score, GW/SLV, SMI, GNRI, donor age, and blood loss. A multivariable analysis based on the significant variables in the univariate analysis revealed that the presence of HAD (hazard ratio [HR] [95% CI: 95-percent confidence interval] 16.54 [3.50–78.06]; p < 0.001) was an independent prognostic factor for all-cause mortality (Table 3).

**Table 3:** The prognostic factors of mortality in LDLT patients in Cox proportional hazard model analyses

Predicter	Unadjusted			Adjusted		
	HR (95% CI)		p-value	HR (95% CI)		p-value
HAD (HAD = 1, non-HAD = 0)	17.43	(3.29–92.46)	< 0.001	16.54	(3.50–78.06)	< 0.001
Age	1.00	(0.94–1.08)	0.891			
Gender (male = 1, female = 0)	5.97	(1.20–29.66)	0.013	5.72	(1.26–26.07)	< 0.01
BMI	0.93	(0.76–1.12)	0.456			
MELD score	0.99	(0.90–1.08)	0.892			
GW/SLV	1.07	(1.00–1.15)	0.046	1.05	(1.00–1.11)	0.055
SMI (low = 1, high = 0)	0.96	(0.87–1.05)	0.365			
GNRI (< 92 = 1, ≥ 92 = 0)	0.28	(0.06–1.39)	0.123			
CKD (CKD = 1, non-CKD = 0)	1.32	(0.38–4.62)	0.664			
Donor age	0.99	(0.95–1.04)	0.797			
Blood loss	1.00	(1.00–1.00)	0.800			

Values were reported as the hazard ratio and 95-percent confidence interval (95% CI)

*BMI* body mass index; *CKD* chronic kidney disease; *95% CI* 95-percent confidence interval; *GNRI* Geriatric Nutritional Risk Index; *GW/SLV* graft weight/standard liver volume; *HAD* hospital acquired disability; *HR* hazard ratio; *LDLT* living donor liver transplantation; *MELD score* model for end-stage liver disease score; *SMI* skeletal muscle index

Furthermore, we also considered the prognostic factors associated with HAD and SMI (Table 4). The significant poor prognostic factors for all-cause mortality were the HAD/low SMI group, gender and GW/SLV. The HAD/low SMI group (HR [95% CI] 16.82 [2.96–95.68]; p = 0.002) was identified as an independent prognostic indicator for all-cause mortality in the multivariable analysis.

**Table 4:** The prognostic factors on mortality in four groups according to HAD and SMI of LDLT patients in Cox proportional hazard model analyses

Predicter	Unadjusted			Adjusted		
	HR (95% CI)		p-value	HR (95% CI)		p-value
Non-HAD and high SMI		[Reference]			[Reference]	
Non-HAD and Low SMI	8.09	–	1.000	6.90	–	1.000
HAD and High SMI	8.05	(1.51–43.06)	0.015	9.18	(1.83–45.99)	0.007
HAD and Low SMI	20.82	(2.85–152.09)	0.003	16.82	(2.96–95.68)	0.002
Age	1.00	(0.94–1.07)	0.922			
Gender (Male = 1, Female = 0)	5.53	(1.08–28.26)	0.023	5.27	(1.16–24.07)	0.012
BMI	0.91	(0.75–1.09)	0.320			
MELD score	1.01	(0.91–1.10)	0.871			
GW/SLV	1.07	(1.01–1.15)	0.031	1.05	(1.00–1.13)	0.049
GNRI (< 92 = 1, ≥ 92 = 0)	0.28	(0.06–1.35)	0.120			
CKD (CKD = 1, non-CKD = 0)	1.39	(0.40–4.85)	0.609			
Donor age	0.99	(0.95–1.04)	0.812			
Blood loss	1.00	(1.00–1.00)	0.457			

Values were reported as the hazard ratio and 95-percent confidence interval (95% CI)

*BMI* body mass index; *CKD* chronic kidney disease; *95% CI* 95-percent confidence interval; *GNRI* Geriatric Nutritional Risk Index; *GW/SLV* graft weight/standard liver volume; *HAD* hospital acquired disability; *HR* hazard ratio; *LDLT* living donor liver transplantation; *MELD score* model for end-stage liver disease score; *SMI* skeletal muscle index

## Discussion

To the best of our knowledge, this is the first study to assess the influence of HAD in LDLT recipients, furthermore, HAD and mortality assessments in LDLT patients are scant in literature. The main findings of the present study were as follows: (1) HAD was identified in approximately 35% of LDLT patients (2); in the HAD/low SMI group, all-cause mortality was the highest, and (3) HAD was an independent risk factor for all-cause mortality, and the HAD/low SMI group had similar outcomes.

Although a different clinical population, Saitoh et al. [23, 35] showed that HAD accounts for approximately 25% of cardiac disease patients. A previous meta-analysis study also reported that HAD occurs in approximately one-third of all hospitalized patients [15]. In our results, above 35% LDLT patients showed HAD, which is consistent with the results of this study. Interestingly, the non-HAD group was also more severely condition with MELD scores, and ADL was significantly more dependent than those in the HAD group. In other words, in the non-HAD group, many preoperative LDLT patients could not independently perform ADL due to poor general condition. However, the Barthel index at hospital discharge in the non-HAD group was not significantly different between the HAD group, indicating that ADL in the HAD group recovered well. Our results suggest that it is important to improve physical function in hospitalized patients.

Improvement in physical activity in solid organ transplantation recipients have shown improved lean mass, muscle strength, and consequently better aerobic capacity, however, little data exist for liver transplant recipients. Berzigotti et al. [36] mentioned dividing the problems into postoperative early and late phases. In the early post-transplantation phase, immobilization is associated with bed rest, extended hospital and intensive care stay, corticosteroid and immunosuppressant drugs-associated myopathy, and episodes of organ rejection. In later phases, calcineurin inhibitor-induced effects (e.g., reduction in mitochondrial respiration and muscle regeneration/remodeling) and metabolic syndrome-related problems are common with post-liver transplantation and contribute to further worsening of aerobic capacity. In addition, he mentioned that only 50% of patients are able to perform regular physical activity within 2 years of liver transplantation, this is one of the potential reasons of failure to reverse muscle loss post-transplantation.

Physical inactivity is clearly associated with obesity, diabetes mellitus, arterial hypertension, coronary heart disease, and osteoarthritis. Skeletal muscle is now recognized as an endocrine organ that secretes cytokines such as myokines and other peptides, which have attracted attention in recent years [37]. Interestingly, myokines are involved in the inflammatory response, and physical activity plays a key role in the anti-inflammatory phenotype homeostasis, and liver function also increases the availability of glucose for uptake and oxidation by myokines [38]. In this way, physical activity has a high potential for providing long-term beneficial effects. Certainly, in our patients, the ADL score was improved at hospital discharge in the HAD group. However, long-term recovery of ADL was unknown because we did not evaluate long-term follow-up. Evidence on the benefits of exercise on clinical outcomes derived from large clinical trials is still missing.

Although all patients also received perioperative rehabilitation, and ADL scores in the non-HAD group were significantly improved from admission to discharge hospital, in our results, the stair and bathing items of the ADL scale were more dependent than other items. Nishiwaki et al. [39] pointed out that stair and bathing items decreased easily in the Barthel index. In particular, stair and bathing were presumed as difficult activities among the ADL assessment tool. Therefore, we were suggested that enhanced rehabilitation programs including these ADL training was need.

The HAD/low SMI group showed the highest all-cause mortality among the groups. Loss of muscle mass, muscle strength, and physical performance are common in patients with chronic liver disease, and are associated with increased all-cause mortality and poor clinical outcomes [40]. Previous studies have shown that similar to pre-transplantation renal dysfunction, SMI was associated with all-cause mortality in chronic liver disease [41–44]. In post-operative liver transplantation patients sarcopenia is thought to be responsible for the decrease in lean body mass, infections, renal dysfunction [45]. Since renal function was not significantly different in this study, we presumed that HAD has a greater effect than renal function. In addition, the use of immunosuppressive agents such as mTOR and calcineurin inhibitors may have an additional role in affecting the skeletal muscle [46]. Although, the most common cause of death is graft failure, regarding direct consequence with long-term physical activity was unknown. In our previous study, LDLT patients with bile duct complications had pre-operative lower SMI, it cannot be denied that troubles due to bile duct complications may have affected the graft function [13]. Our results suggested that differences in ADL improvement during hospitalization may affect ADL after hospital discharge, and factor for predicting prognosis. However, in our results, it was difficult to draw firm conclusions from lack of the physiological basis.

Furthermore, we considered sub-analysis that divided into the low or high SMI group. As a result, no one died in the non-HAD/low SMI group. Although, preoperative intensive rehabilitation intervention is ideal for LDLT patients with poor general condition and physical function, realistically, patients who have difficult rehabilitation intervention. Therefore, postoperative rehabilitation is important in LDLT patients. However, there are patients who responder and non-responder in recovery of ADL with similar rehabilitation programs. Since our subjects excluded in-hospital deaths, LDLT patients with liver function and general condition should be improving at the hospital discharge. In this study, since the causes of death including carcinoma were different respectively, it was difficult to identify the causal relationship with the cause of death. Our results suggest that HAD patients were no significant differences due to the difference in SMI, but preoperative low SMI patients complicated HAD may have a worse prognosis. However, since the number of patients who died also was small, and multiple comparisons reduce the statistical power to detect significance, and further consideration was not possible.

Our previous studies reported that sarcopenia was not related to short-term prognosis of LDLT patients [13, 14]. It is possible that HAD with physical activity is more important than sarcopenia in terms of long-term mortality. Although increased physical activity improves of mortality is speculated, the physiological basis has not been proven in this study, and the results have to be interpreted with caution. Postoperative patients of liver transplantation is presumed that muscle hypertrophy takes a long time due to the promotion of proteolysis associated with inflammation, and the decrease in protein synthesis ability caused by graft failure and underfeeding. We have previously confirmed decreased quadricep muscle thickness by ultrasound after 1 month of LDLT, thus, it may be difficult to prevent skeletal muscle atrophy in such cases as it depends on the recovery of the graft [10]. Although rehabilitation makes muscle hypertrophy difficult in short-term, it is possible to recovery of ADL is relatively easy. Therefore, to prevent HAD, and for proper perioperative management of LDLT patients, rehabilitation should be started as quickly. Early rehabilitation is suggested that increases physical activity which plays a crucial role in the prevention of sarcopenia and HAD, and decrease of all-cause mortality. In addition, several previous studies have suggested that perioperative nutritional therapy improves skeletal muscle mass and mortality, and the aid of pre-transplantation nutritional intervention combined rehabilitation such as comprehensive management might be able to improve outcomes after LDLT [45, 47].

The present study had several limitations. First, the sample size was relatively small. Second, this study was conducted at a single institute, that may have caused selection bias. Third, this was a retrospective study. Lastly, preoperative physical function and performance (e.g., handgrip and quadriceps force, and 6-min walk test) were not evaluated, which could not investigate the relation of physical performance and HAD. Additionally, long-term follow-up of ADL was not done. Large-scale studies including comprehensive evaluation of muscle mass, muscle force, and physical function are needed to evaluate the association of HAD with mortality in LDLT patients.

## Conclusions

This study indicated that HAD could affect prognosis in follow-up patients after LDLT, suggesting that it could be one of the key components in determining prognosis after LDLT. Consequently, it is necessary to initiate increased activity as quickly as possible after LDLT. In the perioperative comprehensive rehabilitation, future larger-scale studies are needed to consider the overall strategy, such as enhanced recovery after surgery (ERAS), including nutritional therapy.

## Supplementary Information

**Additional file 1:** **Table. S1.** Comparison in low SMI of LDLT patients and surgery characteristics.

**Additional file 2:** **Table S2. **Causes of death in LDLT patients.

## Data Availability

The datasets used and/or analyzed during the current study available from the corresponding author on reasonable request.

## Abbreviations

ADL: Activities of daily living
BI: Barthel index
BMI: Body mass index
CKD: Chronic kidney disease
CT: Computer tomography
eGFR: Estimated glomerular filtration rate
GNRI: Geriatric Nutritional Risk Index
GW/SLV: Graft weight/standard liver volume
HAD: Hospital acquired disability
HU: Hounsfield unit
ICU: Intensive care unit
IQR: Interquartile range
LDLT: Living donor liver transplantation
MELD score: Model for end-stage liver disease score
SMI: Skeletal muscle index
