# Supplementary material for: Association between hospital acquired disability and post-discharge mortality in patients after living donor liver transplantation
Source: BMC Surg. 2022 Dec 29;22:445. doi: 10.1186/s12893-022-01896-2 (PMC9798581; doi:10.1186/s12893-022-01896-2)
Supplement: Supplementary file 1 — Additional file 1: Table. S1. Comparison in low SMI of LDLT patients and surgery characteristics. [file 12893_2022_1896_MOESM1_ESM.docx]

**Table. S1** Comparison in low SMI of LDLT patients and surgery characteristics.

|  | Low SMI group | |  | High SMI group | |  |
| --- | --- | --- | --- | --- | --- | --- |
|  | HAD group　(n=12) | non-HAD group (n=31) | p-Value | HAD group　(n=35) | non-HAD group (n=58) | p-Value |
| Gender, Male, n (%) | 7 (58.5) | 15 (48.4) | 0.736 | 18 (51.4) | 33 (57.0) | 0.668 |
| Age, year | 56.0 (46.3 to 63.3) | 57.0 (50.0 to 61.0) | 0.839 | 57.0 (52.0 to 65.0) | 57.0 (52.0 to 63.0) | 0.837 |
| BMI, kg/m^2^ | 20.0 (16.1 to 22.0) | 18.9 (15.8 to 21.0) | 0.551 | 20.1 (17.8 to 22.3) | 20.4 (17.7 to 23.4) | 0.916 |
| GNRI, points | 80.7 (75.2 to 89.1) | 78.7 (69.7 to 85.8) | 0.386 | 84.4 (73.6 to 91.5) | 83.8 (75.5 to 92.2) | 0.797 |
| Type of disease, n (%) |  |  |  |  |  |  |
| Hepatocellular carcinoma | 5 (41.7) | 10 (32.3) | 0.723 | 14 (40.0) | 25 (43.1) | 0.684 |
| Primary biliary cirrhosis | 0 | 1 (3.2) |  | 6 (17.1) | 4 (6.9) |  |
| Primary sclerosing cholangitis | 2 (16.7) | 2 (6.5) |  | 1 (2.9) | 2 (3.5) |  |
| B liver cirrhosis | 0 | 3 (9.7) |  | 4 (11.4) | 8 (13.8) |  |
| C liver cirrhosis | 6 (50.0) | 12 (38.7) |  | 12 (34.3) | 21 (36.2) |  |
| Non-B, non-C liver cirrhosis | 1 (8.3) | 2 (6.5) |  | 5 (14.3) | 6 (10.3) |  |
| Other | 3 (25.0) | 11 (35.5) |  | 7 (20.0) | 17 (29.3) |  |
| Comorbidity, n (%) |  |  |  |  |  |  |
| Chronic kidney disease | 6 (50.0) | 16 (48.5) | 1.000 | 18 (51.4) | 20 (34.4) | 0.277 |
| Pre-operative eGFR, mL/min/1.73m^2^ | 60.2 (39.5 to 81.3) | 61.6 (45.4 to 80.9) | 0.705 | 60.2 (44.1 to 90.5) | 70.1 (55.1 to 89.9) | 0.552 |
| Post-operative eGFR, mL/min/1.73m^2^ | 66.2 (62.2 to 72.8) | 61.1 (45.6 to 79.6) | 0.731 | 51.6 (42.5 to 75.0) | 63.9 (45.9 to 73.9) | 0.276 |
| MELD score | 15.0 (10.3 to 28.0) | 17.0 (12.0 to 18.0) | 0.839 | 15.0 (12.0 to 18.0) | 17.0 (11.0 to 21.3) | 0.495 |
| Operation time, min | 849.0 (751.3 to 962.0) | 739.0 (686.0 to 803.0) | 0.024 | 777.0 (710.0 to 897.0) | 752.0 (702.5 to 829.3) | 0.312 |
| Operative blood loss, g | 7848 (4900 to 13293) | 4900 (3700 to 8900) | 0.053 | 5880 (2930 to 12000) | 5225 (3463 to 8627) | 0.667 |
| Donor age, year | 34 (29.0 to 45.8) | 36.0 (30.0 to 53.0) | 0.898 | 33.0 (26.0 to 39.0) | 31.5 (26.8 to 40.3) | 0.649 |
| Left lobe graft, n (%) | 8 (66.7) | 21 (67.7) | 0.802 | 23 (65.7) | 36 (62.1) | 1.968 |
| GW/SLV, % | 37.8 (33.7 to 45.3) | 42.5 (33.0 to 52.9) | 0.330 | 40.2 (33.7 to 44.4) | 39.7 (33.7 to 51.6) | 0.224 |
| ABO-incompatible, n (%) | 1 (8.3) | 11 (35.5) | 0.059 | 7 (20.0) | 11 (19.0) | 1.759 |
| Initial walking, day.4 | 11.5 (6.3 to 41.3) | 11.0 (7.0 to 15.0) | 0.514 | 13.0 (7.0 to 20.0) | 8.0 (5.0 to 11.3) | 0.002 |
| ICU length of stay, days | 5.5 (5.0 to 15.8) | 5.0 (4.0 to 8.0) | 0.484 | 6.0 (4.0 to 9.0) | 5.0 (3.8 to 7.3) | 0.140 |
| BI at admission, points | 100 (100 to 100) | 100 (65 to 100) | 0.042 | 100 (95 to 100) | 100 (80 to 100) | 0.223 |
| BI at hospital discharge, points | 90 (86.3 to 95) | 100 (100 to 100) | < 0.001 | 90 (70 to 95) | 100 (100 to 100) | < 0.001 |
| Hospital length of stay, days | 58.0 (39.3 to 99.5) | 52.0 (37.0 to 76.0) | 0.515 | 58.0 (47.0 to 76.0) | 42.0 (33.0 to 52.3) | < 0.001 |
| Transfer to hospital, n (%) | 4 (33.3) | 6 (19.4) | 0.427 | 15 (44.1) | 5 (8.6) | < 0.001 |
| Number of patients of 3 years survival | 4 (33.3) | 0 | 0.004 | 8 (22.9) | 2 (3.5) | 0.012 |

**Notes:** Values were reported as the median and Interquartile range (IQR) or number of subjects and percentage. *Significant compared control group p < 0.05. **Abbreviations:** BI: Barthel index; BMI: body mass index; eGFR: estimated glomerular filtration rate; GNRI: Geriatric Nutritional Risk Index; GW/SLV: graft weigh / standard liver volume; HAD: hospital acquired disability; ICU: intensive care unit; IQR: interquartile range; LDLT: living donor liver transplantation; MELD score: model for end-stage liver disease score; SMI: skeletal muscle index.
