# Supplementary material for: Association between hospital acquired disability and post-discharge mortality in patients after living donor liver transplantation
Source: BMC Surg. 2022 Dec 29;22:445. doi: 10.1186/s12893-022-01896-2 (PMC9798581; doi:10.1186/s12893-022-01896-2)
Supplement: Supplementary file 2 — Additional file 2: Table S2. Causes of death in LDLT patients. [file 12893_2022_1896_MOESM2_ESM.docx]

**Table S2** Causes of death in LDLT patients

| n (%) | HAD + Low SMI (n=12) | non-HAD + Low SMI (n=31) | HAD + High SMI (n=34) | non-HAD + High SMI (n=58) |
| --- | --- | --- | --- | --- |
| Graft failure | 1 (8.3%) | 0 | 2 (5.7%) | 0 |
| Infection | 1 (8.3%) | 1 (3.2%) | 1 (2.9%) | 0 |
| Hepatocellular carcinoma | 0 | 0 | 1 (2.9%) | 0 |
| Other carcinoma | 1 (8.3%) | 0 | 2 (5.7%) | 0 |
| Post-transplant lymphoproliferative disorder | 0 | 0 | 1 (2.9%) | 0 |
| Chronic rejection | 0 | 0 | 1 (2.9%) | 0 |
| Cerebral hemorrhage | 1 (8.3%) | 0 | 0 | 0 |
| Total | ４ (33.3%) | 1 (3.2%) | 8 (23.5) | 0 |

**Abbreviations:** HAD, hospital-acquired disability; LDLT, living donor liver transplantation; SMI, skeletal muscle index
